# The complexity of dental anxiety and its association with oral health‐related quality of life: An exploratory study

**DOI:** 10.1111/eos.12907

**Published:** 2022-11-23

**Authors:** Vilde Aardal, Kjersti Berge Evensen, Tiril Willumsen, Vibeke Hervik Bull

**Affiliations:** ^1^ Oral Health Centre of Expertise in Rogaland Stavanger Norway; ^2^ Institute of Clinical Dentistry University of Oslo Oslo Norway

**Keywords:** abuse, anxiety, dental anxiety, oral health, quality of life

## Abstract

This study aimed to explore the factors associated with oral health‐related quality of life (OHRQoL) in a sample with high dental anxiety. Data were obtained from 107 patients attending a therapeutic treatment program for people who have experienced abuse and for those with dental phobia in Norway. Patients completed questionnaires, including the Index of Dental Anxiety and Fear, the Anxiety subscale of the Hospital Anxiety and Depression Scale, and the Oral Impacts on Daily Performance scale, measuring OHRQoL prior to treatment. The various measurement instruments were evaluated psychometrically, and the variables associated with OHRQoL were explored using hierarchical multiple regression. Symptoms of dental anxiety and general anxiety were high, while OHRQoL was poor. Dental anxiety, higher age, higher number of years since the last dental treatment, and higher general anxiety were discernibly associated with lower OHRQoL. The strongest association was found between general anxiety and OHRQoL. In conclusion, several factors were associated with OHRQoL in a sample with high dental anxiety, suggesting a complex picture of dental anxiety. When treating patients with high dental anxiety, dental practitioners should be aware that there may be factors complicating the therapeutic setting, such as general anxiety.

## INTRODUCTION

Quality of life is a broad‐ranging concept defined by the World Health Organization as a person's perception of their position in life relative to their environment [1]. Quality of life includes physical and mental aspects of health, functioning, and well‐being. The impact of oral health on quality of life is described as oral health‐related quality of life (OHRQoL). Several factors may affect OHRQoL in addition to oral function, for example, dental anxiety, age, gender, and previous traumatic experiences [2, 3, 4, 5, 6, 7].

Dental anxiety is an anxiety disorder characterized by a deep, persistent, and unreasonable fear of the dental setting [8]. According to a systematic review from 2021, the global estimated prevalence of high dental fear and anxiety was 15.3% (95% confidence interval [CI] = [10.2, 21.2]) [9]. Dental anxiety may arise as a conditionally learned behavior stemming from previous negative experiences during dental treatment [10] and may develop in childhood, as well as in adolescence or adulthood [11]. For some who develop dental anxiety as children, their anxiety persists; however, many children learn to cope with their fear and are less anxious as they get older [12, 13]. There are also other risk factors not directly connected to the dental treatment situation, indicating that the etiology of dental anxiety is multifactorial [14, 15]. Research suggests that especially people who have experienced some form of trauma or abuse are likely to develop dental anxiety [16, 17]. Elements of the dental setting may remind patients of their trauma experience, such as the administration of sharp objects into the mouth, being horizontally lowered, the smell of dental medications, being alone in a room with a person of authority, anticipating or experiencing pain or judgment, and the feeling of not being in control of the situation [18, 19, 20].

People who suffer from high dental anxiety tend to report low OHRQoL [7, 21]. In 1984, Berggren proposed a model describing dental anxiety as a vicious cycle [22]. According to Berggren's model, a person who suffers from dental anxiety will avoid treatment for a long time, resulting in deterioration of the dentition, a feeling of shame, and a fear of being negatively judged by others, again resulting in an increase or maintenance of the initial anxiety. This model has since been supported by studies suggesting that the consequence of long‐term dental anxiety typically results in the avoidance of dental services, resulting in the requirement of more extensive and complex treatment procedures [23, 24]. In further support of this, Moore et al. [25] found that the number of years spent avoiding the dentist and habits of attempting to hide the mouth from other people were positively correlated with the intensity of embarrassment. Dental anxiety, therefore, may have both physiological and psychological consequences that negatively impact OHRQoL, including painful and uncomfortable oral conditions, as well as poor self‐esteem, a lack of confidence when engaging in social interactions, and fear of judgment [26, 27, 28].

The association between dental anxiety and OHRQoL has attracted increased attention recently. However, to be able to understand this association more thoroughly and understand potential factors influencing this association in different groups of people, more research is needed. The goal of this study was therefore to explore the factors associated with OHRQoL in a sample receiving treatment for high dental anxiety.

## MATERIAL AND METHODS

### Participants and procedures

All participants were recruited from a therapeutic dental service for people who have experienced torture or abuse, and those with dental phobia (abbreviated the TADA service) [29] in Rogaland, Norway, from March 2018 to September 2021. Even though the TADA service aims to include torture survivors, this patient group is underrepresented. Torture survivors were therefore not included in this study. One possible explanation for the underrepresentation of torture survivors, as suggested by Bryne et al. [30], could be the complexity of the migration process.

The TADA service provides cognitive behavioral therapy for people with severe anxiety associated with dental treatment due to previous abuse, and to those who meet the criteria for a dental phobia diagnosis evaluated by a licensed psychologist. Dental phobia is a specific phobia characterized by deep, persistent, and disproportionate fear of the dental setting, and it is a severe form of dental anxiety [8]. The term “dental anxiety” is used throughout this article to capture the entire study sample. All patients were given information about the study over the phone prior to the first appointment. Patients received written information, a consent form, and questionnaires in the mail and were asked to bring the completed questionnaires and signed consent form to the first appointment if they wished to participate in the study. Care was taken to assure the respondents that their treatment in TADA would not be influenced by whether they participated in the study. It was not possible to obtain an exact count of how many patients were included in the TADA service during this period due to a lack of standardized reporting routines, but it is estimated to be between 110 and 150. Patients under 18 years of age and those who could not communicate sufficiently in Norwegian were excluded from the study, in addition to the aforementioned group of torture survivors. One hundred and seven patients were included in the final analysis. The Regional Committees for Medical and Health Research Ethics, West, Norway, approved the study (REC number 241804).

### Measurement instruments

The measurement instruments used in this study were obtained from questionnaires that had already been used in the TADA service nationally in Norway. These questionnaires included questions on sociodemographic variables (age, gender), level of education, questions about the number of years that had passed since the last dental treatment and since the dentition was last fully restored (no further treatment needs), self‐reported inclusion criteria, and standardized instruments designed to measure OHRQoL [31], dental anxiety [32], and symptoms of general anxiety [33].

The OHRQoL was measured using the Norwegian version of the Oral Impacts on Daily Performance (OIDP) scale, which has been validated in the Norwegian population [34]. The OIDP scale is based on a theory about the consequences of oral impacts, and it has been proposed that it measures three different categories of impacts on daily performances, namely physical (eating, speaking, and cleaning teeth), psychological (sleeping/relaxing, smiling, and emotional state), and social (working, and enjoying contact with other people) [31]. This three‐factor structure has since been validated in several studies [35, 36, 37]. However, several studies have also found both one‐factor and two‐factor structure solutions [38, 39]. The OIDP scale consists of eight items and is measured using a five‐point Likert‐type scale (1 = every day or almost every day, 2 = once or twice a week, 3 = once or twice a month, 4 = less than once a month, and 5 = never), with a minimum sum score of 8 and a maximum sum score of 40.

Dental anxiety was measured by using the Index of Dental Anxiety and Fear (IDAF‐4C^+^) [32], which has recently shown good internal consistency in a clinical sample in Norway [40]. The scale consists of a core module, a phobia module, and a stimulus module that can be used together or separately. In this study, the core module was used as a measure of dental anxiety. The core module includes eight items that measure the physiological, emotional, cognitive, and behavioral components related to dental anxiety. Scores are measured on a five‐point Likert‐type scale (1 = disagree, 2 = agree a little, 3 = somewhat agree, 4 = moderately agree, and 5 = strongly agree), resulting in a minimum sum score of 8 and a maximum score of 40. A mean item score of three or above is considered to indicate the level of fear that is a criterion for a specific phobia according to the Diagnostic and Statistical Manual of Mental Disorders (DSM‐IV).

Symptoms of general anxiety were measured using the Norwegian version of the Hospital Anxiety and Depression Scale, Anxiety subscale (HADS‐A), which is a validated subscale questionnaire consisting of seven questions measuring symptoms of anxiety [41]. Each item is scored on a Likert‐type scale from 0 to 3, and the potential scoring outcomes range from 0 (minimum symptoms level) to 21 (maximum symptoms level).

### Statistical analysis

All statistical analyses were performed using IBM SPSS Statistics 28 and IBM SPSS Amos 28. Missing data were excluded pairwise, resulting in some variance in the number of observations for the various statistical analyses. Descriptive analyses were run on the sociodemographic data to obtain frequencies, means, and standard deviations. Then, confirmatory factor analysis (CFA) was run to confirm the factor structures of the instruments measuring OHRQoL, dental anxiety, and general anxiety. Model fit was evaluated using the criteria offered by Byrne et al. [42]. In the case of poor model fit, exploratory factor analysis (EFA) was run using principal axis factoring and direct oblimin rotation. Suitability for EFA was determined by using the criteria offered by Hair et al. [43], Brown [44], and Kline [45]. Kaiser's criterion (the eigenvalues‐greater‐than‐one‐rule) [46] was used to determine the number of factors to retain.

Multiple linear regression analysis was used in a hierarchical manner to explore, step by step, how different variables and factors were associated with OHRQoL. Berggren's vicious cycle of dental anxiety was used as a theoretical framework underpinning the regression modelling. Analyses were assessed for violations of assumptions of normality, linearity, and homoscedasticity [47]. Variance inflation factor (VIF) values, with a set cutoff value at >10, were used to check for multicollinearity [47, 48]. First, the association between dental anxiety (IDAF core module) and OHRQoL (OIDP) was tested. Subsequently, the association between the four factors constituting the IDAF core module (that is, physiological, emotional, cognitive, and behavioral factors) and OHRQoL were investigated to gain a deeper insight into which aspects of dental anxiety were associated with OHRQoL. The significance of the association between the factors and OHRQoL was used as a criterion for deciding whether to keep the factors in subsequent models. The association between background factors—age, gender, a history of abuse—and OHRQoL were tested in the following models before the number of years passed since the last dental treatment was added. Because anxiety disorders often overlap, meaning patients often experience comorbidity of different anxiety disorders [49], general anxiety was added to the model to examine the association with OHRQoL.

The results were reported as adjusted *R* ^2^ values so as not to overestimate the model fit, and statistical significance was determined based on *p*‐values smaller than 0.05. To provide information about the direction of the association as well as the range in which the true value lies, 95% CIs were also reported [50].

## RESULTS

### Sample characteristics and descriptive statistics

A summary of the sample characteristics is depicted in Table 1. The mean age of the participants was 36.0 (SD 9.9) years. More than two‐thirds of the respondents were women, and more than two‐thirds of the respondents had not finished any higher (college/university) education. Approximately 40% reported having experienced abuse. On average, 4.7 (SD 6.7) years had passed since the last dental treatment (*n* = 92), and 9.1 (SD 9.0) years had passed since the dentition was last fully restored (no further treatment needs) (*n* = 70).

**TABLE 1:** Key characteristics of study participants

**Variable**	***n***	**%**
Gender	106	99.1
Female	74	69.2
Male	32	29.9
Unknown	1	0.9
Education	107	100.0
Elementary school 9 years	21	19.6
High school 12 years	54	50.5
Higher education up to 5 years	27	25.2
Higher education 5 years or more	5	4.7
Inclusion criteria	107	100.0
Dental phobia	65	60.7
Abuse	42	39.3

*Note*: The inclusion criterion “abuse” refers to study participants with high dental anxiety or a diagnosed dental phobia who also report having experienced some form of abuse. The inclusion criterion “dental phobia” refers to study participants who have a diagnosed dental phobia, and report no history of abuse

The mean score for the IDAF core module was 33.4 (SD 6.4), and the mean item score was 4.2 (SD 0.8). The mean score for the HADS‐A was 10.0 (SD 4.9). The mean score for the OIDP scale was 26.3 (SD 9.3). Also, 93.4% of participants reported at least one oral impact on daily performance, whereas 6.6% reported no impact (*n* = 91). The daily performances that were most strongly negatively impacted by oral health were eating, enjoying food, smiling, laughing, and showing teeth without embarrassment. Response frequencies are depicted in Table 2.

**TABLE 2:** Distribution of participants according to their responses to each of the items from three measurement instruments—the IDAF core module, OIDP scale, and HADS‐A

**IDAF core module items**	***n***	**Disagree**	**Agree a little**	**Somewhat agree**	**Moderately agree**	**Strongly agree**
I feel anxious shortly before going to the dentist	105	2	4	5	22	72
I generally avoid going to the dentist because I find the experience unpleasant or distressing	106	9	6	10	8	73
I get nervous or edgy about upcoming dental visits	107	2	3	7	15	80
I think that something really bad would happen to me if I were to visit a dentist	102	21	17	19	20	25
I feel afraid or fearful when visiting the dentist	106	2	2	9	20	73
My heart beats faster when I go to the dentist	105	0	3	5	12	85
I delay making appointments to go to the dentist	107	13	4	9	9	72
I often think about all the things that might go wrong prior to going to the dentist	106	16	17	19	11	43

**OIDP scale items**	***n***	**Never**	**Less than once a month**	**Once or twice a month**	**Once or twice a week**	**Every day or almost every day**
Eating and enjoying food	99	16	14	12	18	39
Speaking and pronouncing clearly	101	54	14	11	6	16
Cleaning teeth	100	31	17	11	15	26
Sleeping and relaxing	98	30	19	24	12	13
Smiling, laughing, and showing teeth without embarrassment	100	20	15	8	12	45
Maintaining a balanced emotional state without getting angry	96	30	24	22	10	10
Enjoying contact with other people	97	35	20	17	8	17
Performing daily activities	97	50	17	14	7	9

**HADS‐A items**	***n***	**0**	**1**	**2**	**3**	‐
I feel tense or “wound up”	84	10	39	21	14	‐
I get a sort of frightened feeling, as if something awful is about to happen	93	24	30	25	14	‐
Worrying thoughts go through my mind	91	9	23	25	34	‐
I can sit at ease and feel relaxed	90	18	36	26	10	‐
I get a sort of frightened feeling, like “butterflies” in the stomach	92	19	48	20	5	‐
I feel restless as I have to be on the move	92	17	35	28	12	‐
I get sudden feelings of panic	89	18	38	19	14	‐

Abbreviations: IDAF, Index of Dental Anxiety and Fear; OIDP, Oral Impacts on Daily Performance; HADS‐A, Anxiety subscale of the Hospital Anxiety and Depression Scale.

### Factor analyses

CFA was run on all three measurement instruments. The IDAF core module [32] and HADS‐A [33] showed acceptable and good model fit, respectively. The IDAF core module showed an acceptable model fit for the four‐factor solution it was designed to measure in the CFA, with a non‐significant chi‐square statistic (minimum discrepancy per degrees of freedom [CMIN/DF] = 1.62, *p* = 0.07), a root mean square error of approximation (RMSEA) value of 0.076, a comparative fit index (CFI) of 0.98, and a Tucker‐Lewis index (TLI) of 0.96. Factor loadings on respective factors ranged from 0.75 to 0.95, suggesting convergent validity. Bivariate correlations between factors ranged from 0.26 to 0.81, which was considered an indicator of discriminant validity [44]. Cronbach's alpha values ranged from 0.77 to 0.92, indicating internal consistency reliability for all factors.

The CFA of the HADS‐A showed a good model fit for a one‐factor solution, with a non‐significant chi‐square test statistic (CMIN/DF = 0.97, *p* = 0.48), a RMSEA value of < 0.001, and CFI and TLI values of 1.0. Factor loadings ranged from 0.53 to 0.81, suggesting convergent validity, and Cronbach's alpha was 0.87, suggesting internal consistency reliability.

The OIDP scale did not show an optimal model fit for the suggested three‐factor solution, with a significant chi‐square test (CMIN/DF = 1.74, *p* = 0.3), and a RMSEA value of 0.083. All prerequisites for EFA were met, and EFA was therefore conducted, revealing a unidimensional factor solution explaining 56.1% of the variance. Factor loadings ranged from 0.62 to 0.88, suggesting convergent validity. Cronbach's alpha value of 0.91 indicated internal consistency reliability.

### Associations with oral‐health‐related quality of life (OHRQoL)

The sequential multiple regression analyses exploring the factors associated with OHRQoL are presented in Table 3, together with the standardized beta coefficients, adjusted *R* ^2^ values, and significance levels. VIF values were well below the cutoff, with values ranging from 1.1 to 3.6. The level of dental anxiety was negatively associated with OHRQoL (95% CI = [–0.9, –0.3], *p* < 0.001) (Model 1). The investigation of the various factors of dental anxiety revealed that the behavioral component showed a significant association with OHRQoL (95% CI = [–2.6, –0.9], *p* < 0.001), in contrast to the physiological component (95% CI = [–1.7, 3.2], *p* = 0.5), the emotional component (95% CI = [–2.4, 1.5], *p* = 0.7), and the cognitive component (95% CI = [–1.0, 0.4], *p* = 0.4) (Model 2), and therefore only behavioral dental anxiety was kept in the subsequent models. Higher age (95% CI = [–0.4, –0.4], *p* = 0.02) but not gender (95% CI = [–4.0, 4.4], *p* = 0.9) was associated with reduced OHRQoL in Model 3, where background factors were checked. Having experienced abuse was modestly associated with OHRQoL (95% CI = [–7.5, 0.9], *p* = 0.1) (Model 4). A higher number of years since the last dental treatment was negatively associated with OHRQoL in a model including both behavioral dental anxiety and background factors (95% CI = [–0.6, –0.04], *p* = 0.09) (Model 5). Finally, higher general anxiety was negatively associated with OHRQoL (95% CI, = [–1.1, –0.4], *p* < 0.001) (Model 6). The strongest negative association in the final model was between general anxiety and OHRQoL.

**TABLE 3:** Results of the hierarchy of multiple linear regression analyses to determine the association between oral health‐related quality of life (Oral Impacts on Daily Performance [OIDP] scale), and dental anxiety (Index of Dental Anxiety and Fear [IDAF] core module), age, gender, abuse experience, years passed since last dental treatment, and general anxiety symptoms (Hospital Anxiety and Depression Scale, Anxiety subscale [HADS‐A])

	**Model 1**	**Model 2**	**Model 3**	**Model 4**	**Model 5**	**Model 6**
Variables	β	β	β	β	β	β
IDAF core module	–0.41^**^					
IDAF core module divided into factors						
Physiological dental anxiety		0.11				
Emotional dental anxiety		–0.08				
Behavioral dental anxiety		–0.50^**^	–0.42^**^	–0.39^**^	–0.33^**^	–0.24^*^
Cognitive dental anxiety		–0.10				
Age			–0.26^*^	–0.23^*^	–0.22^*^	–0.26^**^
Gender			0.01	0.06	0.02	0.05
Abuse experience				–0.18	–0.19	–0.11
Years since last dental treatment					–0.19	–0.23^*^
HADS Anxiety						–0.41^**^
*R* ^2^‐adjusted	0.16	0.21	0.27	0.29	0.31	0.46
*F* for change	16.48^**^	6.84^**^	9.43**	2.50	2.92	19.34^**^

^*^ *p* < 0.05; ^**^ *p* < 0.01.

## DISCUSSION

In this study, we found that higher levels of dental anxiety, higher age, a longer period of avoiding dental visits, and higher levels of general anxiety were negatively associated with OHRQoL in a sample of patients in a treatment program for people with high dental anxiety, suggesting a complex picture of dental anxiety.

First, some limitations of this study need to be addressed. The number of participants was relatively low, which increases the risk of type II errors [51]. However, the population from which the study sample was recruited is small, and the response rate was high. This is also an under‐researched area, which justifies a small‐scale study [52]. Another limitation of this study was the absence of oral health measures. It would be beneficial, in future studies, to have either clinical measures of oral status, or a measure of self‐perceived oral health. The mean sum score for the OIDP scale may indicate that the oral health in this sample is poor; however, this should be interpreted with caution, and research, including clinical measures, is needed. Only one of the factors constituting the IDAF core module was associated with OHRQoL, namely behavioral dental anxiety. This may indicate that the measurement instrument is not sufficiently sensitive to capture how symptoms of dental anxiety possibly associate with OHRQoL. A broader scale with more items and a scale with more response options should probably be used in future research to examine all aspects of dental anxiety more closely.

Using Berggren's vicious cycle as a theoretical framework, we hypothesized that high dental anxiety and delayed dental visits would be associated with OHRQoL. As expected, both dental anxiety and the length of time passed since the last dental treatment were negatively associated with OHRQoL. When investigating the various factors of dental anxiety separately, only behavioral dental anxiety showed a discernible association with OHRQoL. The behavioral element of dental anxiety is related to the avoidance of dental treatment, which, according to Berggren [22], exaggerates the problem by feeding into the vicious cycle of dental anxiety, leading to the deterioration of oral health, a feeling of embarrassment, and a further strengthening of the initial anxiety. Higher age was negatively associated with this sample's OHRQoL. This finding is contrary to studies of both the general Norwegian and Swedish adult populations, which found that higher age had a positive effect on one or more dimensions of OHRQoL [2, 3]. However, Schuller et al. [53] found that older people with high dental anxiety had significantly fewer remaining teeth than those with low dental anxiety.

The levels of general anxiety scores in our sample were high, and also markedly higher than the general Norwegian population [54]. Interestingly, general anxiety had the strongest association with OHRQoL, even stronger than the association between dental anxiety and OHRQoL, although our sample comprised patients seeking treatment for dental anxiety. To the best of our knowledge, this is the first study to show the relationships between general anxiety and dental anxiety and OHRQoL. However, a recent study by Hajek and König [55] found a negative association between generalized anxiety and OHRQoL in the general German population, supporting our findings

The recommended therapy for phobic disorders, including dental anxiety, is exposure therapy [56]. However, a recent review shows that general anxiety is an inhibitor to experiencing the effect of exposure therapy [56]. Our findings may therefore indicate that therapy should be shifted from mere exposure therapy to a more holistic therapeutic approach for patients with high levels of dental anxiety. This is supported by findings from other recent studies on dental anxiety treatment. For example, in a qualitative study on patients with high dental anxiety, patients emphasized the practitioner's attitude and appearance in the therapeutic situation, rather than exposure therapy and techniques, when asked about what was important for alleviating anxiety symptoms [57]. In a randomized controlled study, Hauge et al. [35] found that using midazolam (a benzodiazepine) in treating dental anxiety was as effective as exposure therapy, which may indicate that the dentist's attitudes and the reception of the patients are more important than the therapeutic techniques used in alleviating concrete phobias. Similar results were found by Willumsen et al. [58], comparing cognitive therapy, applied relaxation, and nitrous oxide sedation in treating dental fear.

To conclude, our findings complement and add to previous literature suggesting that dental anxiety is a complex phenomenon. The role of general anxiety as a driving force in the vicious cycle for patients with high and long‐standing dental anxiety should be further investigated.

## AUTHOR CONTRIBUTIONS

**Conceptualization**: Vilde Aardal, Kjersti Berge Evensen, Tiril Willumsen, Vibeke Hervik Bull. **Methodology**: Vilde Aardal, Kjersti Berge Evensen, Tiril Willumsen, Vibeke Hervik Bull. **Formal analysis**: Vilde Aardal, Kjersti Berge Evensen, Vibeke Hervik Bull. **Investigation**: Vilde Aardal, Kjersti Berge Evensen. **Writing—original draft preparation**: Vilde Aardal, Kjersti Berge Evensen, Vibeke Hervik Bull. **Writing—review and editing**: Vilde Aardal, Kjersti Berge Evensen, Tiril Willumsen, Vibeke Hervik Bull.

## FUNDING INFORMATION

This study was funded by the Oral Health Centre of Expertise in Rogaland, Norway.

## CONFLICTS OF INTEREST

The authors report no conflicts of interest.
